# Expression of two barley proteinase inhibitors in tomato promotes endogenous defensive response and enhances resistance to *Tuta absoluta*

**DOI:** 10.1186/s12870-018-1240-6

**Published:** 2018-01-25

**Authors:** Rim Hamza, Meritxell Pérez-Hedo, Alberto Urbaneja, José L. Rambla, Antonio Granell, Kamel Gaddour, José P. Beltrán, Luis A. Cañas

**Affiliations:** 1 Instituto de Biología Molecular y Celular de Plantas (CSIC-UPV). Ciudad Politécnica de la Innovación Edf, 8E. Av. Ingeniero Fausto Elio sn, 46022 Valencia, Spain; 2 Universitat Jaume I (UJI). Departament de Ciències Agràries i del Medi Natural, Unitat Associada d’Entomologia UJI-IVIA, Campus del Riu Sec, E-12071 Castelló de la Plana, Spain; 3 Instituto Valenciano de Investigaciones Agrarias (IVIA). Centro de Protección Vegetal y Biotecnología, Unidad Asociada de Entomología UJI-IVIA, Carretera CV-315, Km 10,7, 46113 Moncada Valencia, Spain; 4 Research Unit of Genome, Immunodiagnostics and Valorization, ISBM, University of Monastir, Monastir, Tunisia

**Keywords:** Proteinase inhibitors, *Tuta absoluta*, Enhanced resistance, Induced plant defense

## Abstract

**Background:**

Plants and insects have coexisted for million years and evolved a set of interactions which affect both organisms at different levels. Plants have developed various morphological and biochemical adaptations to cope with herbivores attacks. However, *Tuta absoluta* (Meyrick) (Lepidoptera: Gelechiidae) has become the major pest threatening tomato crops worldwide and without the appropriated management it can cause production losses between 80 to 100%.

**Results:**

The aim of this study was to investigate the in vivo effect of a serine proteinase inhibitor (BTI-CMe) and a cysteine proteinase inhibitor (Hv-CPI2) from barley on this insect and to examine the effect their expression has on tomato defensive responses. We found that larvae fed on tomato transgenic plants co-expressing both proteinase inhibitors showed a notable reduction in weight. Moreover, only 56% of these larvae reached the adult stage. The emerged adults showed wings deformities and reduced fertility. We also investigated the effect of proteinase inhibitors ingestion on the insect digestive enzymes. Our results showed a decrease in larval trypsin activity. Transgenes expression had no harmful effect on *Nesidiocoris tenuis* (Reuter) (Heteroptera: Miridae), a predator of *Tuta absoluta,* despite transgenic tomato plants attracted the mirid. We also found that barley cystatin expression promoted plant defense by inducing the expression of the tomato endogenous wound inducible *Proteinase inhibitor 2* (*Pin2*) gene, increasing the production of glandular trichomes and altering the emission of volatile organic compounds.

**Conclusion:**

Our results demonstrate the usefulness of the co-expression of different proteinase inhibitors for the enhancement of plant resistance to *Tuta absoluta*.

**Electronic supplementary material:**

The online version of this article (10.1186/s12870-018-1240-6) contains supplementary material, which is available to authorized users.

## Background

Since the beginning of plants domestication, thousands of years ago, pests have been the major threat for agriculture. Nowadays, around 40% of worldwide crop production is destroyed by pests and pathogens, with 13% due to insect attacks [1].

To cope with the broad variety of phytophagous insects, we need to strengthen plant defense arsenal. Plant genetic engineering provides access to a countless number of genes with the potential to improve resistance toward pests. Currently, the most used insecticidal genes are *Bacillus thuringiensis (Bt) Cry* coding Bt toxins. However, efficiency of Bt toxins is limited to a narrow range of insects. Indeed, many Bt pro-toxin molecules require proteolytic activation after solubilization in the gut of the susceptible insect. The use of plant defensive secondary metabolites, like proteinase inhibitors (PIs), is probably the most promising alternative [2, 3]. Plant PIs are small proteins, mostly found in seeds, that are expressed in vegetative tissues in response to wounding, enhancing plant resistance to insects [4, 5].They affect the growth and development of a wide range of insects. Plant PIs have previously been introduced into different plant species conferring efficient pest resistance [6–8]. However, in a co-evolving system, insects adapt to the host plant PIs by synthesizing enzymes of other families which are insensitive to the host PIs [9, 10]. This enzymatic response has been observed within the proteinase classes, showing that one serine proteinase can be substituted by another [11, 12].The combined use of serine and cysteine PIs in artificial diets has shown a synergistic effect on *Tribolium castanum* (Herbst.) (Coleoptera: Tenebrionidae) [13]. To avoid insect adaptation, it has been suggested to select PIs from non-host plants. Indeed, it has been shown that insects feeding on dicots cannot adapt to PIs from monocots and vice versa [14, 15].

Cereals are known for their high content in PIs. In barley, different serine and cysteine proteinase inhibitors have been identified. The serine proteinase inhibitor BTI-CMe shows a high inhibition of trypsin-like activity and it has been successfully used to improve resistance toward different pests [16, 17]. On the other hand, the cysteine proteinase inhibitor Hv-CPI2 shows a high inhibition of papain and cathepsin-L-like activity in vitro [18]. We selected both PIs as candidates for the improvement of tomato plants resistance against *Tuta absoluta*.

Tomato (*Lycopersicon esculentum*) represents, currently, the second most important vegetable crop [19]. Its production reaches 100 million tons of fresh fruit cultivated on 3.7 million hectares. The availability of its genome sequence [20], together with the efficient genetic and genomic resources, allows the development of new genotypes to give response to the customer, producer and processor needs. Different transformation protocols mediated by *Agrobacterium tumefaciens,* using leaves or cotyledons, have been developed [21–24]. Micro-Tom is a miniature cherry tomato cultivar characterised by a short life cycle. Its dwarf genotype is primarily conferred by two recessive genes [25]. Micro-Tom has some interesting traits such as its reduced size, short life cycle (70–90 days from sowing to fruit-ripening) and small genome (950 Mb). Therefore it is currently considered a model cultivar for tomato functional genomics and genetics.

When facing abiotic or biotic stress, tomato plants activate a series of defensive mechanisms. Phytophagous attack or mechanic wounding activates a 18 amino acid peptide called systemin [26]. This protein induces the octadecanoid pathway leading to the synthesis of jasmonic acid, hormone that is responsible of the activation of different direct and indirect defensive mechanisms. Jasmonic acid induces both local and systemic accumulation of proteinase inhibitors [27], volatile organic compounds synthesis [28, 29] and glandular trichomes differentiation [30–32]. The most studied proteinase inhibitor in tomato is PIN2, a trypsin and chymotrypsin inhibitor [33]. PIN2 expression in tobacco and poplar reduced respectively *Manduca sexta* (L.) (Lepidoptera: Sphingidae) growth [34] and *Plagiodera versicolora* Laicharting (Coleoptera: Chrysomelidae) weight and development [35]. Pest challenged tomato plants also alter their volatile organic compounds (VOCs) in order to repel attackers or attract natural enemies [36, 37]. Some of these compounds are synthesized in glandular trichomes. These hairy structures act as chemical and mechanical barrier against pests [38, 39]. Insect damage has been negatively associated with trichomes production [40–42], while other studies have shown that foliage consumption by insects was reduced in plants with high trichomes density [43, 44].

One of the most harmful tomato pests is the South American tomato pinworm *Tuta absoluta* (Meyrick) (Lepidoptera: Gelichiidae). This tomato borer was first described in South America and, in the last decade, has invaded to most of Europe, Africa and Asia [45]. If no control measures are taken, this insect might cause up to a total crop loss [46]. The larval instars are harmful. Indeed, after hatching, young larvae penetrate leaves, fruit and stems and spend the major part of the four instars growing and feeding inside the plant, and therefore hindering the access of insecticides. Currently, the control strategies against this pest are mainly based on chemical treatments and biological control using zoophytophagous mirids such as *Nesidiocoris tenuis* (Reuter) and *Macrolophus pygmaeus* Rambur (Hemiptera: Miridae)*,* which feed both on *Tuta absoluta* eggs and young larvae [47, 48].

In the last two decades, different reports have paid attention to the effect of genetically engineered plants harboring PIs genes on insects. However, none has investigated the effect that the expression of these PIs could have on the host plant endogenous defensive mechanisms. In this study we examined the effect of the expression of two proteinase inhibitors, belonging to different families, against *T. absoluta* and its impact on the endogenous defensive response in tomato plants. We report that the co-expression of both proteinase inhibitors had an additive effect and enhanced tomato plants resistance against *T. absoluta*. Moreover, Hv-CPI2 induced the endogenous defensive mechanisms of the tomato plants.

## Methods

### Plant material and growth conditions

Barley (*Hordeum vulgare* cv. Rihane) seeds were germinated in the dark on vermiculite substrate at 25–30 °C (day) and 18–20 °C (night) and were irrigated daily with Hoagland’s solution [49].

Tomato plants (*Solanum lycopersicum* cv. Micro-Tom; IBMCP seed collection, Spain), were grown in pots with coconut fiber at 25–30 °C (day) and 18–20 °C (night) and were irrigated daily with Hoagland’s solution [49]. Osram lamps (Powerstar HQI-BT, 400 W) were used to supplement natural light in order to get a 16 h light photoperiod.

### Bacterial strains and media

*Escherichia coli* strains DH5α and DH10B were used for gene cloning. *Agrobacterium tumefaciens* strain LBA4404 was used for tomato *Agrobacterium*-mediated transformation. Both strains were grown on LB medium at 37 °C and 28 °C respectively under agitation (200 rpm). *Agrobacterium* growth media was supplemented with 100 mg/l spectinomycin and 100 mg/l rifampicin (final concentration in media). Spectinomycin at 100 mg/l was used for DH10B strain, and ampicilin 100 mg/l (final concentration in media) for DH5α strain. X-Gal at 20 mg/l (final concentration in media) was used for both *E. coli* strains.

### RNA extraction and gene isolation

Barley 12 days old etiolated leaves were collected and used for RNA extraction and cDNA synthesis. Total RNA was extracted using the EZNA Plant RNA Kit (OMEGA bio-tek) and the genomic DNA was eliminated by the Turbo DNase (Ambion) according to the manufacturers’ instructions. Reverse-transcription was realized using the Primer Script RT reagent kit (TaKaRa). The coding sequences for the BTI-CMe and Hv-CPI2 mature proteins were amplified by PCR with the primer couples: CMeS /CMeAS and CPI2S/CPI2AS respectively. The obtained fragments were cloned in the pGem -T -easy vector (Promega) and sequenced to confirm their identity. All the primers used are presented in Additional file 1.

### Genetic constructs

*Itr1* and *Icy2* genes coding respectively for BTI-CMe and Hv-CPI2 were both cloned in the binary vector pK2GW7.0 [50], downstream of the CaMV 35S promoter by the Gateway cloning technology (Invitrogen). To generate the double transgene expression cassette, the coding region of *Itr1* and *Icy2* genes, as well as the 35S promoter and the 35S terminator were first cloned separately in the pGem-T-easy vector (Promega), then, combined in the pCR8/GW/TOPO vector (Thermo Fisher). The cassette was later on transferred to the plant transformation vector pK2GW7.0. *Agrobacterium tumefaciens* strain LBA4404 was transformed with the three obtained constructs.

### Tomato genetic transformation

To generate transgenic tomato plants in the Micro-Tom cultivar, we followed the method previously described by Ellul et al. [21]. In this protocol, cotyledons from germinated seeds (10 days) are used as starting material, followed by co-culture with *Agrobacterium tumefaciens* (strain LBA4404) and using the neomycin phosphotransferase (*nptII*) marker gene to carry out selection of transformants in a kanamycin medium. Tomato plants (*Solanum lycopersicum* cv. Micro-Tom) were transformed with the three constructs previously generated. Ten days old cotyledons were sectioned at its edges and incubated with the recombinant *Agrobacterium* strain in presence of 200 μM acetosyringone to promote bacteria virulence, during two days in the dark at 24 ± 2 °C. Explants were then washed with medium supplemented with 300 mg/l cefotaxime to eliminate *Agrobacterium* excess, and placed on organogenesis medium without antibiotic. Two days later, the explants were transferred to a selective medium containing 100 mg/l kanamycin. Explants first produced calli that differentiated to shoots. About 6–8 weeks later regenerated plantlets were transferred to the rooting medium containing 100 mg/l of kanamycin. When roots reached about 1 cm in length, the plantlets were transferred to soil conditions and acclimated. The selective pressure was maintained during the whole in vitro process to avoid false positives. After acclimation, the ploidy level of the plants was checked by flux cytometry, and the presence of the expression cassette verified by PCR of the *nptII* gene (primers Kan-dir and Kan-rev, Additional file 1). The primary transformants were self-fertilized to produce the T1 generation. Heterozygous lines with a single copy of the transgene were selected by segregating seeds on kanamycin added medium. Homozygous lines were obtained by germination of T2 seeds on selective medium.

### Analysis of gene expression levels

Semi-quantitative PCR was achieved using the CMe-S and CMe-AS primers for the *Itr1* gene and CPI2-S and CPI2-AS primers for *Icy2* (Additional file 1).

Quantitative Real Time PCR (qPCR) was carried out using SYBR Green PCR Master Mix kit (Applied Biosystems) and the 7500 Fast Real-Time PCR System (Applied Biosystems) on 1 μg of cDNA. Data analysis was performed using System Sequence Detection Software v1.2 (Applied Biosystem). Each sample was processed in triplicate. Relative expression levels were determined using the housekeeping gene *SlActin8* [51] as a reference gene using the ΔΔCt method (Applied Biosystems). The primers used in the qRT-PCR are qItr1-F and qITR1-R for *Itr1* gene, qIcy2-F and qIcy2-R for *Icy2* gene and qPIN2-F and qPIN2-R for Tomato *Pin2* gene (Additional file 1).

### *Tuta absoluta* feeding trials

The experiments were conducted on *T. absoluta* from the colony reared in the department of Plant Protection and Biotechnology, at the Valencian Institute for Agriculture Research (IVIA, Valencia). Three *T. absoluta* couples were placed with wild type tomato plants. Two days later, eggs were collected. Twenty individual leaves from each transgenic line and a wild type control, were placed in petri dishes on 2% agar. One single *T. absoluta* egg was deposited on each leave, and the development of the hatched larvae was followed daily. Leaves were renewed every two days. Plates were incubated at 24 ± 2 °C with a photoperiod of 16 h of light/ 8 h darkness. Larvae were also weighted, twenty four hours after each molting. The duration of the larval instars, as well as the entire developmental cycle, were registered for each insect.

### Oviposition assays

The adults emerged from the larvae fed on either transgenic or control plants, were collected and sexed according to the abdomen shape and color. Male adults present a thinner and darker abdomen [52]. Five couples were randomly formed from the emerged adults of each plant type. They were, then, transferred to plastic cups (370 cm^3^) carrying a fresh tomato apical flush. According to the methodology described by Mollá et al. [53], the plastic cups were placed into small ones (230 cm^3^) containing water. The tomato flush reached the water through a hole made in the inner cup. The bigger cup was covered with a fine muslin cloth and fixed with a rubber band. Forty eight hours later, the tomato flush was removed and the number of deposited eggs was counted under a steromicroscope.

### Overall toxicity evaluation

To estimate the combined effect of mortality and oviposition reduction on *T. absoluta* population, we calculated the reduction coefficient E based on the corresponding reduction values (RV) using the Abbot formula [54]. The Reduction coefficient can only be calculated when there is a statistically significant difference. Therefore it was only estimated for CMe-CPI.3.

$$ \mathrm{Rv}\ \mathrm{survival}=\frac{\% Control Survival-\% Experiment Survival}{\% Control Survival\ } $$

$$ \mathrm{Rv}\ \mathrm{fecundity}=\frac{\left(\mathrm{Eggs}\ \mathrm{per}\ \mathrm{Female}\right)\ \mathrm{Control}-\left(\mathrm{Eggs}\ \mathrm{per}\ \mathrm{Female}\right)\ \mathrm{Experiment}}{\left(\mathrm{Eggs}\ \mathrm{per}\ \mathrm{Female}\right)\ \mathrm{Control}} $$

$$ \boldsymbol{E}=100\mathrm{x}\ \left[1-\left(\mathrm{RVsurvival}\ \mathrm{x}\ \mathrm{RVfecundity}\right)\right] $$

### Enzymatic assays

Approximately 40 mg of larvae of the four instars from each treatment were pooled and ground in liquid nitrogen. The powder was mixed with 200 μl of ice cold extraction buffer (0.1 M Tris pH 7, 0.1% ascorbic acid, 0.1% L-cysteine, 0.5 M sucrose and 10 mg/ml PVP). The tubes were centrifuged at high speed for 15 min at 4 °C and the supernatant recovered and mixed with two volumes of ice cold 90% acetone. The mixture was then incubated for 2 h at − 20 °C, and centrifuged at high speed at 4 °C during 10 min. The pellet was washed twice by 90% acetone, dried and re-suspended in 100 μl of 0.5 M Tris buffer pH 8. The obtained crude extract was used to determine both trypsin and papain activity. Nα-benzoyl-L-arginine 4-nitroanilide hydrochloride (BapNa, Sigma) was used as a chromogenic substrate for trypsin and pGlu-Phe-Leu p-nitroanilide (PFLNA, Sigma) as a substrate for papain. The trypsin-like and papain-like activity in the sample was determined by using a gradient of a commercial trypsin (bovine trypsin, Sigma) and papain (Sigma) as standards.

The protein concentration of the crude extract was measured by the Bradford method [55]. Briefly, 5 μg of proteins of the crude extract were mixed with 5 μl of the corresponding substrate (10 mg/ml) and up to 100 μl Sodium phosphate buffer 67 mM pH 7.6 with 20 mM CaCl_2_ for trypsin assays or 5 mM L-cysteine for papain assays. Each sample was incubated in duplicate at 37 °C for 30 min, and absorbance measured at 405 nm. As standards, we used commercial trypsin and papain at six known concentrations (0.125 μg, 0.25 μg, 0.5 μg, 0.75 μg, 1 μg and 1.5 μg). Trypsin and papain activity was expressed as the percentage of trypsin-like or papain-like proteins from the sample’s total protein content.

### Enzyme histochemistry

The fluorescent substrate Nα-benzoyl-L-arginine-7-amido-4-methylcoumarin hydrochloride (BAAMC, Santa Cruz Biotechnology), specific to trypsin and papain was used to localize the targeted proteases in the insect. Larvae of the third instar, fed with wild type plant leaves, were sacrificed by freezing in liquid nitrogen, then included in the cry-protector gel NEG-50 (Richard-Allan Scientific) and frozen at − 27 °C. Cryo-sections of 16 μm were realized with the cryostat (HM520 Microm). Sections were recovered on a poly-lysine coated slide and washed with 10% polyvinyl alcohol (PVA) in PBS 67 mM pH 7.6 to avoid macromolecules diffusion. Then, 50 μl of substrate solution (10% PVA, 0.5 μl BAAMC 20 mg/ml, 2 mM CaCl_2_ in PBS 67 mM pH 7.6) was applied to the section. The slide was incubated at 37 °C for 15 min and then washed 5 times for 1 min in 5% PVA in PBS 67 mM pH 7.6 and once with PBS 67 mM pH 7.6. Sections treated with BAAMC were examined for fluorescence using ultraviolet light with a Leica DM5000 microscope.

### *Nesidiocoris tenuis* Feeding assay

Five plants of the CMe-CPI.3 transgenic line and wild type Micro-Tom tomato were placed in individual cages (bugdorm) with three couples of *N. tenuis* each. *Nesidiocoris tenuis* individuals were provided by Koppert Biological Systems, S.L. (Águilas, Murcia, Spain). The colony of *N. tenuis* was maintained in climatic chamber at 25 ± 2 °C, 60–80% RH and 16:8 h (L:D) photoperiod 25 ± 2 °C, 60–80% RH and 16:8 h (L:D) photoperiod at IVIA. This colony was caged on tomato plants with access to *Ephestia kuehniella* Zeller eggs (Entofood®; Koppert B.S.) as supplemented food until used in bioassays. Five day old adults of *N. tenuis* were used in all the experiments.

In the feeding assay, *N. tenuis* were provided, as alternative food, *E. kuehniella* eggs ad libitum. The different plants were checked every two days, from eggs hatching to adults’ emergence. Nymphal developmental time and the number of adults emerged were recorded.

### Olfactory response

The behavioral response of *T. absoluta* and *N. tenuis* adults to the transgenic plants CMe-CPI.3 volatiles was investigated according to the protocol described by Pérez-Hedo et al. [48] in a Y- shaped tube. The base of the tube was connected to an air pump providing a unidirectional airflow. The side arms were connected to two glass jars each one containing a different odor source: transgenic or wild type plant. Each container was connected to a flow meter and a water filter. For each experiment, 40 adults for both species; 20 females and 20 males were tested. Each insect was observed until it reached at least 3 cm up one of the side arms of the tube or until 10 min have passed [48]. The insects that had not chosen any arm after 10 min were considered as “non responders” and were discarded from the analysis. After five individuals were tested, the olfactometer tube was reverted to minimize spatial effect of arm choice, and after each 10 insects, the odor source was changed.

### Volatile organic compounds analysis

Volatiles organic compounds (VOCs) collection was performed according to the protocol described by Bouagga et al. [56]. VOCs were captured on a headspace solid-phase micro extraction (HS-SPME). Separation and detection were performed by means of gas chromatography coupled to a mass spectrometer (GC/MS). Fibers were mounted on a SPME fiber holder and injected trough the first septum of the sample container. The fiber was extended by pushing the plunger of the SPM filter holder and exposed to plant volatiles. For each plant, volatiles adsorption was performed during 3 h. Each treatment had 6 replicates. After volatiles adsorption, the fiber is drawn back into the needle and the SPME device removed. Desorption was performed in a 6890 N gas chromatograph coupled to a 5975B mass spectrometer (Agilent Technologies). Chromatograms were processed using the Enhanced ChemStation E.02.02 software (Agilent Technologies).

Comparison of both retention time and mass spectrum with those of pure standards allowed the identification of the compounds. All the standards were purchased from Sigma-Aldrich. For quantitation, one specific ion was selected for each compound, followed by the integration of the corresponding peak area from the extracted ion chromatogram. Ions were selected for the highest signal-to-noise ratio and the specificity in that chromatogram particular region in order to provide accurate peak integration.

### Trichomes density determination

The fourth and the fifth leaves of young tomato plants were collected. Both adaxial and abaxial leaf surfaces were examined under an optical microscope (Leica 5000) and glandular trichomes counted.

### Statistical analysis

Statistical analysis was realized with the Graph Pad Prism 6 software. Duration of developmental instars were analyzed by ANOVA test, while larval weight and oviposition, for each transgenic line, were compared to wild type plants by t test. Chi-square tests of independence were applied to compare survival percentage and olfactory response.

## Results

### *Agrobacterium-*mediated transformation of tomato plants and transgene expression analyses

**Fig. 1:**
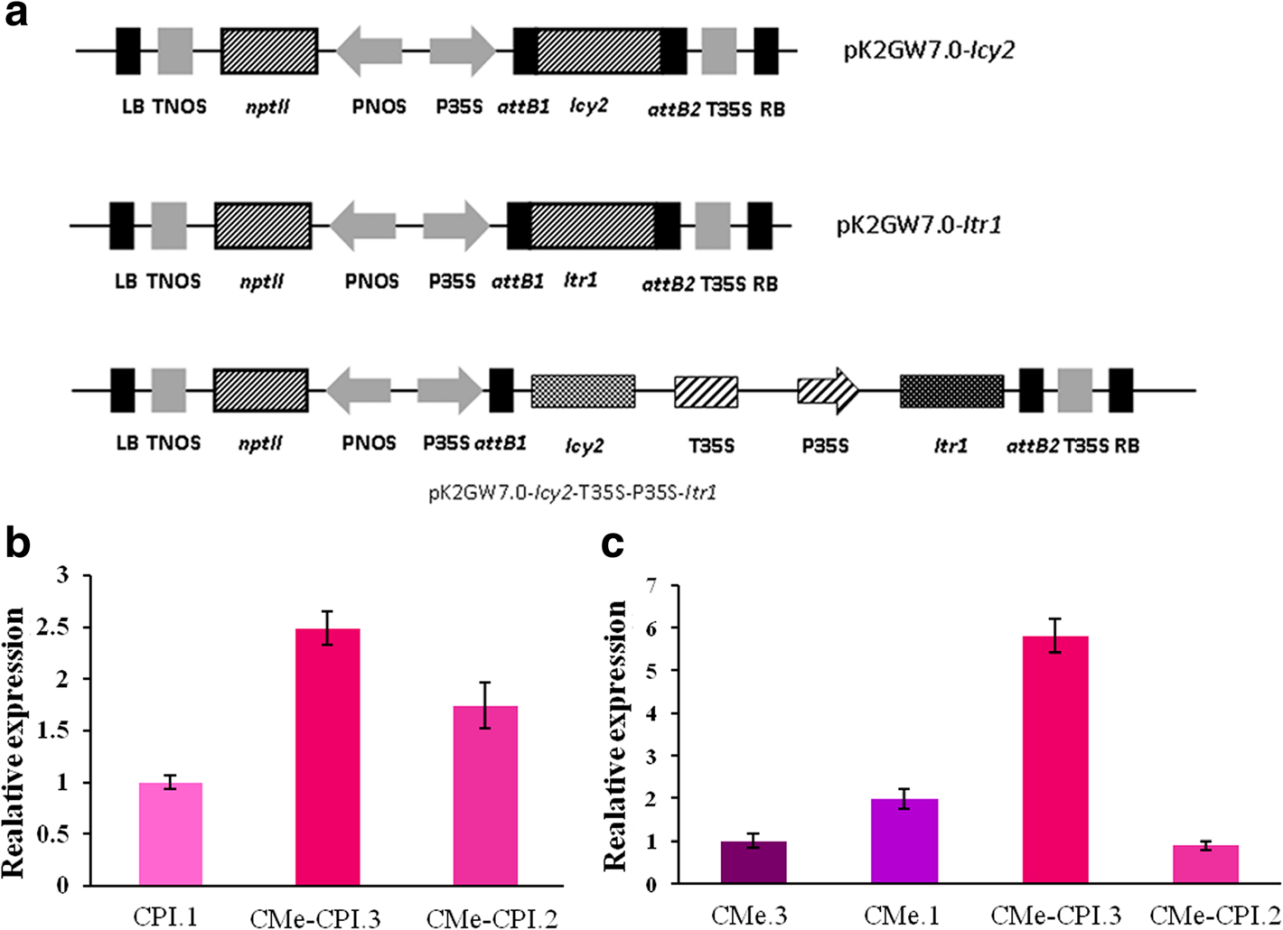
Genetic constructs and relative expression of *Icy2* and *Itr1* genes in the different transgenic lines. **a** Genetic constructs used for *Agrobacterium*-mediated transformation of tomato. **b** Relative expression of *Icy2*. **c** Relative expression of *Itr1*. CMe-CPI.3 shows the highest expression level of both transgenes. CMe.1 and CPI.1 have the highest expression level of *Itr1* and *Icy2*, respectively

Tomato explants were cocultured with *A. tumefaciens* strain LBA4404 carrying the three constructs containing the BTI-CMe and Hv-CPI2 transgenes (Fig. 1a). After co-cultivation, callus started to be formed at the cut ends of about 60% of the explants. Six transgenic independent lines (T0) were obtained from plants expressing *Itr1* and *Icy2*, and another eight plant lines expressing both transgenes were isolated. All these transgenic plants were diploid.

Five homozygous transgenic lines expressing *Itr1*, three expressing *Icy2* and six lines expressing both transgenes were retained for further characterization. Transgenic plants expressing *Itr1* were named “CMe”, plants expressing *Icy2* were named “CPI” and plants co-expressing both proteinase inhibitors were named “CMe-CPI”.

With the aim to select the transgenic lines expressing the highest levels of the transgenes, we first performed a semi-quantitative RT-PCR (Additional file 2). Lines showing the highest transgene expression level were submitted to a qRT-PCR assay for more sensitive analyses. qRT-PCR amplification measurements indicated that there are no endogenous *Itr1* or *Icy2* genes in tomato, therefore proving the expression of the transgenes had no internal interferences in the analyzed plants. According to the qRT-PCR measurements, among the transgenic lines expressing *Icy2* individually, CPI.1 showed the highest transgene expression level. Among plants expressing *Itr1*, CMe.1 was the line with highest expression of the transgene. CMe-CPI.3 was the double transgenic line showing the highest expression level for both transgenes, *Itr1* and *Icy2*. CMe-CPI.3 expressed the *Itr1* gene 2.9 times more than CMe.1 line and the gene *Icy2* 2.5 times more than CPI.1 line (Fig. 1b-c). These three transgenic lines, CPI.1; CMe.1, and CMe-CPI.3 were selected to carry out insect feeding trials.

### Insects feeding trials

Feeding *T. absoluta* with CMe-CPI.3 transgenic plants affected the insect at different levels; larval weight, survival and fecundity. As it can be seen in Table 1, a slight delay in the first larval developmental instar was observed on larvae fed with leaves of the CPI.1 transgenic plant. Insects fed with the other transgenic lines showed no significant differences when compared with the wild type.

**Table 1:** Larval developmental instars of *Tuta absoluta* fed with leaves of transgenic and wild type plants

	1st instar (days)	2nd instar (days)	3rd instar (days)	4th instar (days)	Total development
CMe-CPI.3	3.71 (*n* = 14, *p* = 0.053)	3.63 (*n* = 11, *p* = 0.630)	2.18 (n = 11, *p* = 0.302)	3.00 (n = 11, *p* = 0.997)	12 (*n* = 10, *p* = 0.069)
CMe.1	3.62 (*n* = 13, *p* = 0.123)	3.33 (*n* = 12, *p* > 0.999)	2.08 (n = 12, *p* = 159)	2.44 (*n* = 9, *p* = 0.320)	11.75 (n = 8, *p* = 0.370)
CPI.1	3.80 (*n* = 15, ***p*** **= 0.023**)	3.06 (*n* = 15, *p* = 0754)	2.00 (n = 14, ***p*** **= 0.033**)	2.27 (n = 11, *p* = 0.196)	11.27 (n = 11, *p* = 0.999)
WT	3.07 (n = 14)	3.21 (n = 14)	2.57 (n = 14)	2.83 (n = 14)	11.25 (n = 12)

Few days delay in the first instar is observed on larvae fed with leaves of the CPI.1 transgenic plant. In larvae fed with leaves of the other transgenic lines no significant difference was observed. *p* values in bold indicate significant differences

**Fig. 2:**
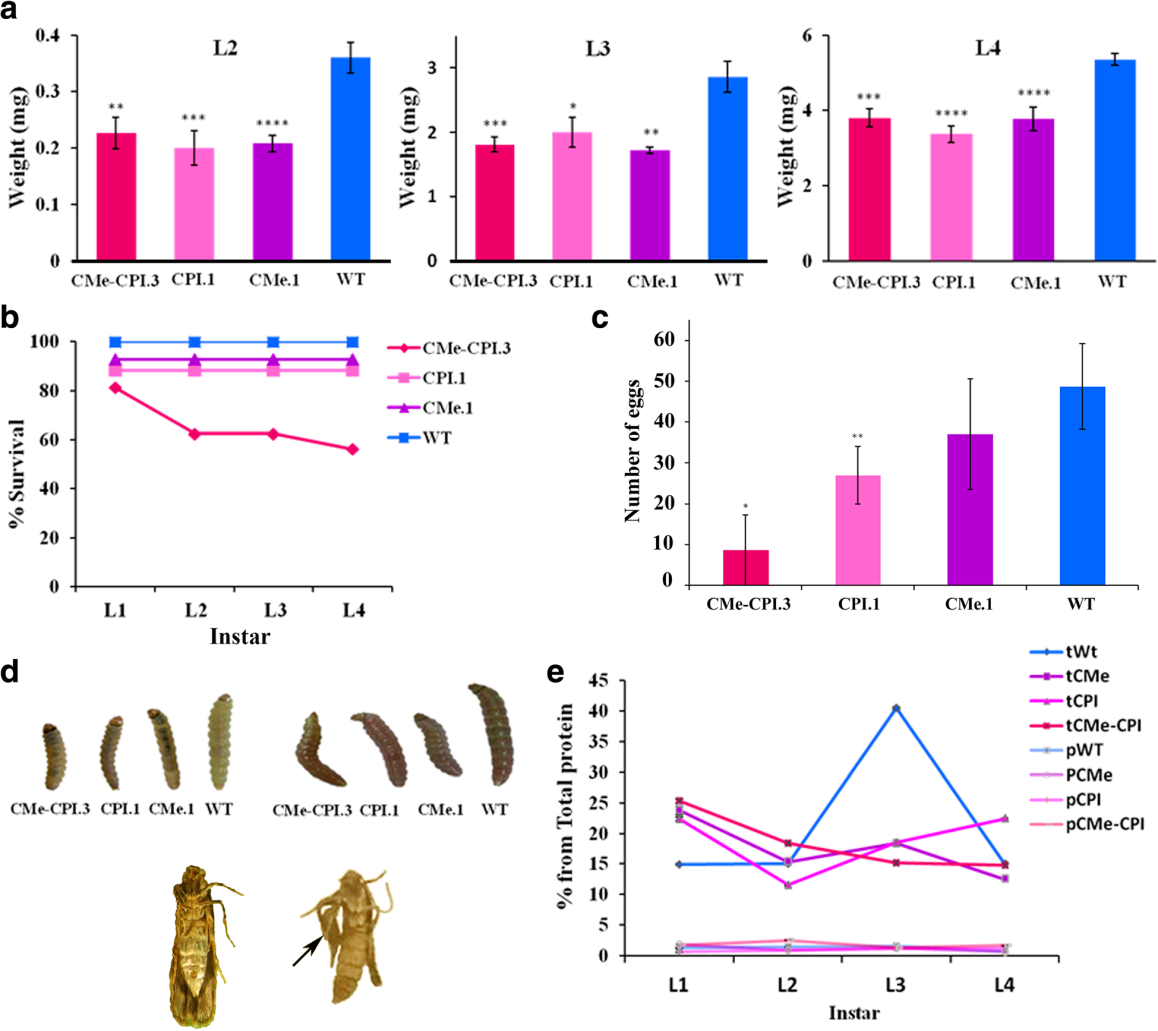
*Tuta absoluta* feeding trials. **a** Larvae weight when fed with the three transgenic plants and the wild type. Larval weight is reduced in all larvae fed with the transgenic leaves. Statistical test: t test, *n* = 8, significance *p* < 0.05. **b** Larval survival decreased with the increasing number of feeding days reaching 56.25% (Chi test, *p* = 0.018) for the CMe-CPI.3 transgenic line. The first and second instar showed the highest mortality level. **c** Number of eggs laid after 48 h. Decrease in the number of eggs for adults emerged from larvae fed on the different transgenic lines, mostly CMe-CPI3. (*n* = 5, *t* = 2.54, df = 7, *p* = 0.022). **d** Morphological alterations. Upper left: L3 larvae fed with transgenic and wild type plants. Larvae fed with the three transgenic plants show reduced size; Upper right: L4 larvae fed with transgenic and wild type plants; larvae fed with the three transgenic plants show reduced size; Bottom left: Adult female emerged from a larva fed with WT plants; Bottom right: Wing deformity observed in a female adult emerged from a larva fed with transgenic plants (arrow). **e** Trypsin and papain activity in insects’ crude extract. Trypsin-like and papain-like activity decay in larvae fed with the different transgenic plants when compared with wild type. t: refers to trypsin and p: refers to papain

Feeding *T. absoluta* with transgenic plants affected larval weight and size at all larval instars. In the first instar, larval weight of insects feeding on transgenic plants could not be determined with the balance (weights below 0.1 mg). As shown in Fig. 2a, in all the other instars the larvae fed on transgenic leaves presented a lower weight than those fed with the wild type ones.

Mortality of larvae was observed during the four instars (Fig. 2b). Larvae survival decreased to 56.2% (Chi square, *p* = 0.01) when they fed on CMe-CPI.3 transgenic plants. The first and second instars showed the highest mortality level.

We counted the deposited eggs of couples previously fed, during larval stages, with leaves of the different transgenic and wild type plants. As shown in Fig. 2c, a reduction in the number of laid eggs was observed when adults emerged from larvae were fed with the different transgenic lines, mostly with the CMe-CPI.3 line (*n* = 5, *t* = 2.54, df = 7, *p* = 0.022) and CPI.1 line (n = 5, t = 2.54, df = 7, *p* = 0.019). Around 40% of the adults emerged from larvae fed with leaves of CMe-CPI.3 transgenic plants showed wings deformities (Fig. 2d) (Chi = 4.8, df = 1, *p* = 0.02). It is worthy to mention that these individuals were unable to mate, and subsequently to lay eggs. The reduction coefficient (E) obtained after the Abbot correction, reflecting the combined effect of mortality rate and reduced fecundity was 64%.

Feeding *T. absoluta* with the three types of transgenic plants reduced significantly the trypsin activity of the insect. The protein extracts from larvae of the four instars were tested for cysteine proteinase and trypsin-like activities. The cysteine proteinase activity, in larvae of *Tuta absoluta* was very low, below 2% of the total proteins compared with trypsin-like activity (12–40%) (Fig. 2e). When the larvae were fed with wild type plants, the trypsin activity was stable during the two first instars (about 15% of total proteins), then showed a considerable increase on the third instar reaching up to 40% before decreasing to about 15% at the fourth instar. The increase of proteolytic activity could be explained by the augment of feeding and the high gain of size and weight of the insect at this instar.

Trypsin activity in larvae fed with leaves from CPI.1 and CMe.1 transgenic plants was relatively high at the first instar (22–23%) then decreased at the second instar (11–15%). This activity increased slightly at the third instar reaching about 18%, but stayed considerably low when compared to the activity in larvae fed with wild type plants. At the fourth instar the trypsin activity continued increasing in larvae fed on CPI.1 plants, while it decreased back in those fed on CMe.1.

Trypsin activity in larvae fed with CMe-CPI.3 leave was about 25% at the first instar and decreased along the increase in the number of feeding days, showing no increment of activity at the third instar (about 17%) (Fig. 2e).

Enzyme histochemistry permitted to localize trypsin-like enzymes in *T. absoluta* L3 larvae. The fluorescence signal was detected at different anatomical levels: the insect digestive system (esophagus, foregut, midgut, hindgut), the exoskeleton and the secretory system (Malpighi tubules), (Additional file 3).

**Fig. 3:**
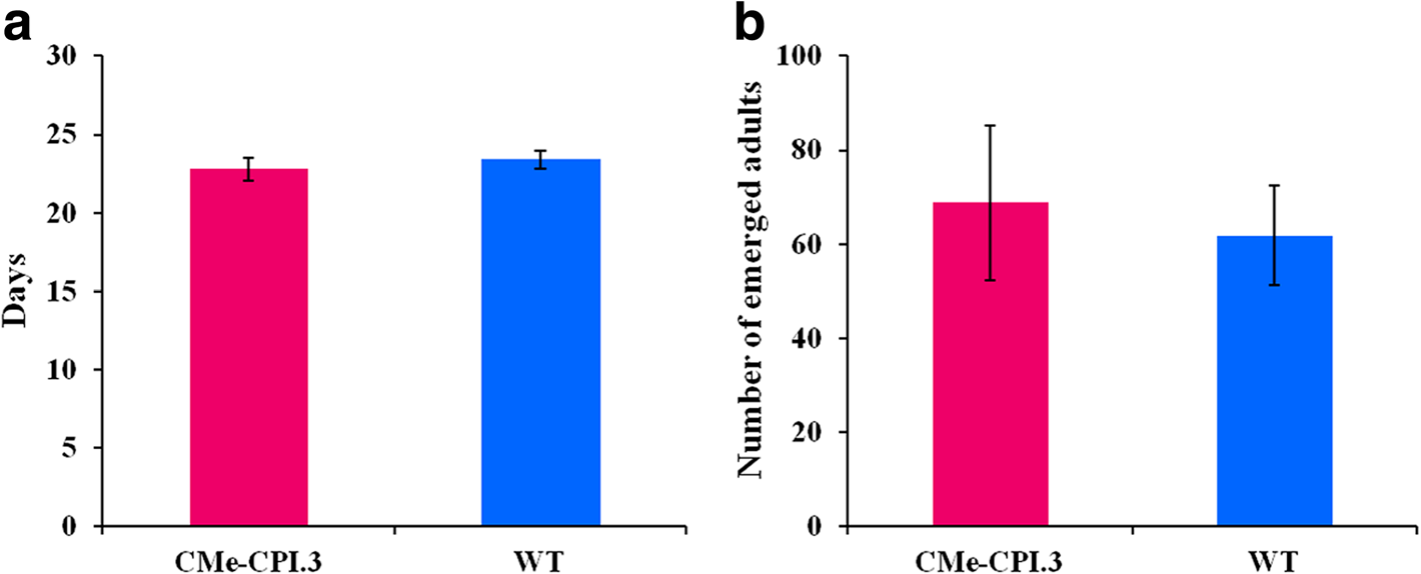
Effect of PIs on the development of *Nesidiocoris tenuis.* **a**. Developmental cycle duration of *Nesidiocoris tenuis* on CMe-CPI.3 and wild type plants; the presence of the PIs does not affect the developmental time of *Nesidiocoris* nymphs (*t* = 0.84, df = 6, *p =* 0.27). **b** Number of *Nesidiocoris tenuis* adults emerged after developing on CMe-CPI.3 and wild type plants (*t* = 0.35, df = 6, *p =* 0.11). No differences were observed between the transgenic and the wild type plants

*N. tenuis* development and survival were not affected by the transgenic plants. *N. tenuis* adults were placed on both CMe-CPI.3 and wild type Micro-Tom tomato plants. The development of the laid eggs was followed until the emergence of adults. The duration of the developmental cycle was the same in the presence of transgenic or wild type plants. In both assays, adults started to emerge 21 days after the beginning of the experiment (*t* = 0.84, df = 6, *p =* 0.27). They were collected and counted and no significant differences in numbers were observed (*t* = 0.35, df = 6, *p =* 0.11), (Fig. 3).

### Tomato endogenous defensive response analyses

CMe-CPI.3 transgenic plants showed an activation of the endogenous defensive response at different levels: tomato PI induction; VOCs profile alteration, and an increment of glandular trichome production.

**Fig. 4:**
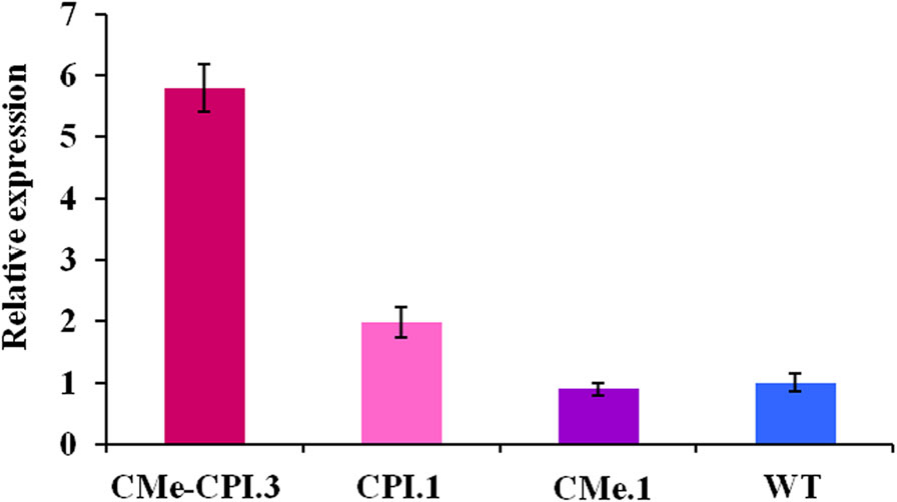
Relative expression of *Pin2* in the different transgenic lines and the wild type Micro-Tom plants. *Pin2* expression was increased in plants expressing *Icy2* (CPI.1 and CMe-CPI.3), while no difference between CMe.1 and the wild type plants was observed

We quantified the expression of the tomato wound-induced proteinase inhibitor PIN2 in the three transgenic lines as well as in the wild type plants. The qRT-PCR measurements revealed that *Pin2* expression was increased in plants expressing the transgene *Icy2*. Indeed, CMe-CPI.3 and CPI.1 plants expressed *Pin2* about six times more and twice more, respectively, than the wild type plants. However, the transgenic plants expressing only *Itr1* transgene did not show alterations in *Pin2* expression level (Fig. 4).

**Fig. 5:**
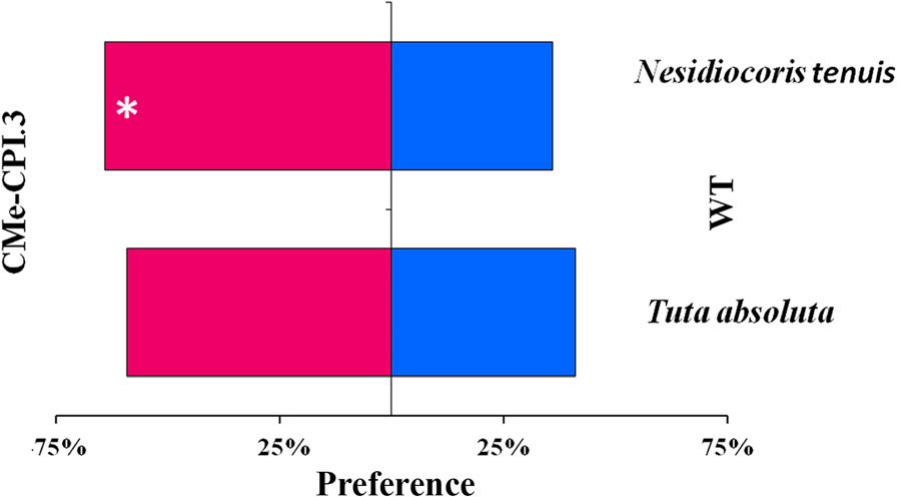
Olfactory response of *T. absoluta* and *N. tenuis* adults to CMe-CPI.3 and wild type tomato volatiles. *Nesiodiocoris tenuis* adults are attracted by CMe-CPI.3 volatiles (Chi = 4.9, df = 1, *n* = 40, *p =* 0.01), while *Tuta absoluta* makes no difference between both plants (Chi = 4.9, df = 1, n = 40, *p =* 0.09)

The olfactory responses of *T. absoluta* and *N. tenuis* to the CMe-CPI.3 and wild type tomato plants were tested*. Tuta absoluta* adults showed no preference to neither transgenic nor wild type plants (Chi = 4.9, df = 1, *n* = 40, *p =* 0.09). However, *Nesidiocoris tenuis* preferred the CMe-CPI.3 transgenic plants to the wild type ones (Chi = 4.9, df = 1, n = 40, *p =* 0.01). When *N. tenuis* was allowed to choose between both kinds of tomato plants, over 63% of the individuals were attracted by the transgenic plants, while about only 36% selected the wild type ones (Fig. 5a).

VOCs emission profile of the transgenic plants CMe-CPI.3 differed from the wild type ones. VOCs from wild type and transgenic CMe-CPI.3 plants were analyzed by GC-MS. Volatile compounds from different chemical families were differentially produced in both plants. When compared with the wild type, CMe-CPI.3 plants showed different levels of benzenoids and terpenes. Benzaldehyde and another unknown benzenoid were emitted twice more in the transgenic plants, while monoterpenes (unknown monoterpene, α-pinene, camphene, β-myrcene, β-pinene) and three unknown sesquiterpenes were reduced to a third part (Table 2).

**Table 2:** Relative changes of the volatiles emitted by wild type and CMe-CPI.3 transgenic plants. Student’s t-test (*p* < 0.05)

Type	Compound	Kovats RI	Fold change	*p* value
Monoterpene	Unknown Monoterpene 1	939.2	0.31	**0.0289**
Monoterpene	α-pinene	948.1	0.28	**0.0245**
Monoterpene	Camphene	969.0	0.35	**0.0428**
Monoterpene	β-myrcene	991.3	0.31	**0.0225**
Monoterpene	β-pinene	996.5	0.30	**0.0330**
Sesquiterpene	Unknown Sesquiterpene 1	1356.4	0.29	**0.0167**
Sesquiterpene	Unknown Sesquiterpene 2	1360.4	0.28	**0.0105**
Sesquiterpene	Unknown Sesquiterpene 3	1417.1	0.42	**0.0378**
Sesquiterpene	β-caryophyllene	1464.1	0.63	0.0823
Benzenoid	Benzaldehyde	976.9	2.12	**0.0004**
Benzenoid	Unknown benzenoid 1	1058.0	1.71	**0.0030**
Benzenoid	Acetophenone	1089.2	3.07	0.0902

CMe-CPI.3 plants emitted less terpenes and higher levels of benzenoids than wild type plants. *p* values in bold indicate significant differences

**Fig. 6:**
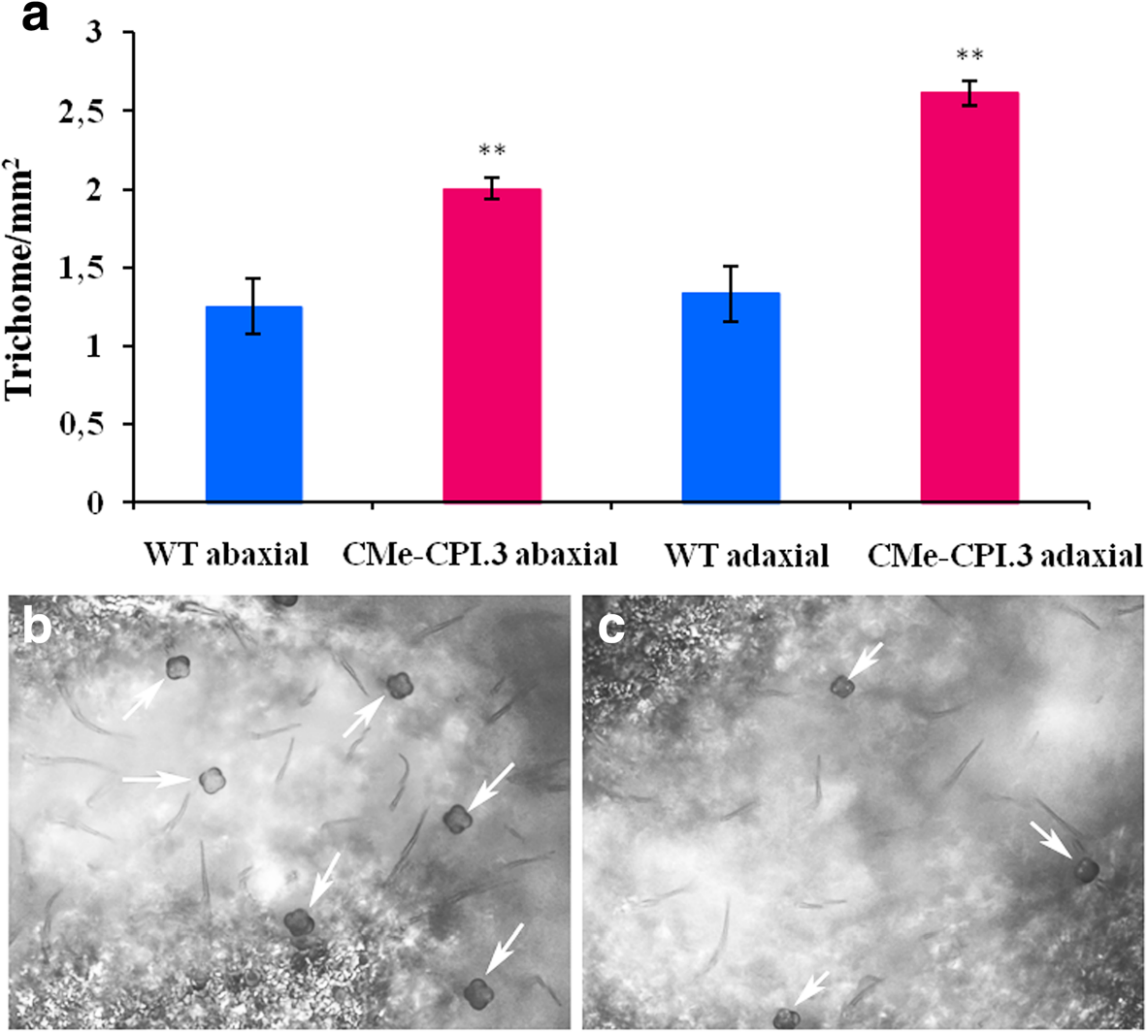
Trichomes density in transgenic and wild type plants. **a** Trichomes density is higher in CMe-CPI.3 transgenic plants leaves when compared with the wild type ones. **b** Transgenic CMe-CPI.3 plants (left) showed higher glandular trichomes density in the abaxial side than wild type plants (right)

The leaves of wild type plants and CMe-CPI.3 transgenic tomato plants were examined under the microscope on both adaxial and abaxial sides. Transgenic leaves showed an increase in glandular trichomes density. The adaxial side of the transgenic leaves presented 1.96 times more glandular trichomes (*t* = 6.56, df = 4, *p* = 0.001), and the abaxial side 1.6 times more (*t* = 3.925, df = 4, *p* = 0.008) (Fig. 6a-b).

## Discussion

### Expression of BTI-CMe and Hv-CPI2 enhances tomato resistance to *T. absoluta*

To induce resistance to *Tuta absoluta* in tomato plants we followed a general strategy proposed by Duan et al. [14], consisting in the expression of proteinase inhibitors (PIs) isolated from monocots in dicots plants, since insects feeding on dicots would be more sensitive and unable to adapt to PIs from monocots and vice versa. We have shown that the co-expression of two proteinase inhibitors from barley, BTI-CMe and Hv-CPI2, in transgenic tomato plants enhances the resistance to the tomato pest *T. absoluta* while attracting its predator *N. tenuis*. The protective effect of BTI-CMe and Hv-CPI2 was observed when both PIs were expressed together.

Insect feeding experiments showed that *T. absoluta* larval weight and survival were reduced when feeding with the transgenic tomato plants compared with the wild type ones. Mean weight loss and survival were respectively 34.2% and 56.3% for CMe-CPI.3; 37.2% and 13% for CMe.1 and 37.2% and 11.8% for CPI.1. Weight loss and mortality can be explained by the inhibitory activities of the PIs against *T. absoluta* digestive enzymes. No previous study has identified *T. absoluta* digestive proteases. In this work, we detected trypsin activity in all larval instars. Also, we were able to localize these proteases by enzymatic histochemical techniques in the different parts of the digestive system (esophagus, foregut, midgut and hindgut), the excretory system (Malpighi tubules) and the exoskeleton. However, almost no papain activity was detected. These findings are in agreement with previous reports which suggested that Lepidoptera digestion relies mainly on serine proteinases [57–59]. Trypsin activity drastically decreased in larvae fed with the three different transgenic tomato plants. The observation that this activity was affected in larvae fed with CPI.1 plants suggests that the detrimental effect observed is not due to cysteine proteinase inhibitory activity of Hv-CPI2.

*T. absoluta* fecundity was also affected, in addition to the reduction in size and survival of the insect. Around 40% of the adults that emerged from larvae fed with CMe-CPI.3 showed wing deformities and were unable to copulate, producing no offsprings. Most individuals did not present morphological deformities although they showed low fecundity. On the other hand, the number of eggs layed by the females was reduced by 82.3%. Similar results were reported on *Helicoverpa armigera* (Hübner) and *Spodoptera litura* (Fabricius) (Lepidoptera: Noctuidae) [60, 61] when using non host PI from bitter gourd and Capsicum, respectively. Tomato PI also affected notably the fecundity of *H. armigera* according to Damle et al. [62]. The fecundity of Lepidoptera adults is an important parameter for determining the effect of larval diet on the adult stage. Also, low fecundity values means less progeny and therefore have a direct impact on the subsequent generation. Considering both insect survival and fecundity data, our experimental approach showed a *T. absoluta* total population reduction value (E) of 64%.

These results show the potential of both PIs when expressed together to enhance tomato resistance against *T. absoluta* and to reduce its population. Previous studies have shown that the effect of PIs on other insects is dose dependent so that higher resistance is acquired when PIs are expressed at high levels [63, 64]. Therefore, the use of genetically engineered tomato plants expressing higher levels of *Itr1* and *Icy2* could inflict stronger harm to *T. absoluta* and provide a better control of its population.

### Expression of BTI-CMe and Hv-CPI2 in tomato had no harmful effects on *Nesdiocoris tenuis*

To check the compatibility of these transgenic plants with current biocontrol strategies in use, we investigated their effect on a *T. absoluta* predator: *Nesidiocoris tenuis*. *N. tenuis* reared on CMe-CPI.3 tomato plants were not affected by the presence of PIs in the plants. No effect was observed neither on their developmental time nor their viability. *Nesidiocoris tenuis* needs to feed on plant material to develop, hence our results suggest that its digestive enzymes are not affected by PIs in the plants.

### Hv-CPI2 expression induces tomato defensive response

The effect of PIs expression in transgenic plants on phytophagous insects has been largely studied, however the effect on the host plant endogenous defense mechanisms have not been investigated. Our results suggest that the expression of the barley cysteine proteinase inhibitor Hv-CPI2 in tomato activates both endogenous direct and indirect defense mechanisms.

Expression analysis of the tomato proteinase inhibitor *Pin2* showed that it is induced in presence of Hv-CPI2. This unexpected induction of the tomato trypsin and chymotrypsin inhibitor could explain both, the inhibitory effect of CPI.1 plants and the synergetic effect observed in CMe-CPI.3 tomato plants. In fact, CMe-CPI.3 over-expresses three PIs belonging to different mechanistic classes: 2 trypsin inhibitors (BTI-CMe and PIN2), a cystatin (Hv-CPI2) and a chymotrypsin inhibitor (PIN2). The co-expression of these three PIs makes *T. absoluta* adaptation more difficult and improbable. It has been documented that some insects are able to overcome the presence of trypsin inhibitors in their diet by shifting the biosynthesis of one type of proteases to another one. Oppert et al. [10] have reported that the red flour beetle, *Tribolium castaneum* (Herbst) (Coleoptera: Tenebrionidae), when fed with cystatin supplemented diet, produces serine proteinase digestive enzymes as a compensatory response. The same phenomenon was observed in *Helicoverpa zea* Boddie (Lepidoptera: Noctuidae), where the presence of the soybean trypsin inhibitor was compensated by the production of chymotrypsins inhibitors [9]. It would be difficult for *T. absoluta* larvae during their short larval development period to achieve a compensatory mechanism toward three PIs of different families.

PIN2 is highly expressed in tomato trichomes both constitutively and in response to herbivores attack [65]. Trichomes production is usually constitutive; however, some plant species increase trichome density in new leaves upon damage [66, 67]. CMe-CPI.3 plants showed higher glandular trichomes density when compared with wild type plants. This finding agrees with previous studies. Luo et al. [68] have shown that the expression of the night shade (*Solanum americanum*) *SaPIN2* gene increased glandular trichomes density in tobacco and enhanced its resistance toward the larvae of the two lepidoptera species pest *H. armigera* and *S. litura* [68]. It has been shown that, when fed with induced leaves, insects consumed less foliage and grown less compared to those fed with non-induced ones [69]. Tomato plants have both non glandular and glandular trichomes. While the first ones act as a mechanical barrier against pests, the second type is responsible for the secretion of a variety of metabolites and volatiles which can be harmful or repellent to insects and/or attractant to their natural enemies [70].

As trichomes are responsible for the production of volatile organic compounds (VOCs), we investigated plant volatiles production and insect olfactory responses. *N. tenuis* adults were attracted by CMe-CPI.3 transgenic plants volatiles, while *T. absoluta* had no preference for either of the two plant lines. These results were supported by the VOCs analysis. CMe-CPI.3 transgenic plants have shown increased levels of benzenoids and reduced levels of monoterpenes and sesquiterpenes when compared with the wild type plants. Benzenoids have previously been described as insect attractants. They have, thus, been reported to attract natural enemies of plant pests. Octyl benzaldehyde was shown to attract *Oryus tristicolor* (White) (Hemiptera: Anthocoridae) (a bug predator of *Tetranychus urticae* Koch. (Acari: Tetranychidae) and thrips (Thysanoptera: Tripidae)) [71]. In addition to the attraction of natural enemies, benzenoids also act as repellents of phytophagous pests. Sesame plant, *Sesamum indicum* L., which represents a natural refuge for mirids shows a stronger attraction for *N. tenuis* than tomato. Naselli et al. [72] have associated this attraction to a reduction of the levels of hydrocarbon monoterpenes. These results agree with our findings: the fact that the CMe-CPI.3 plant secretes relatively low concentrations of hydrocarbon monoterpenes (α-pinene, β-mycene, β-pinene) and high levels of benzenoids could explain its attraction for *N. tenuis* adults.

Terpenoids are synthesized through two pathways: the mevalonic (MVA) and the methylerythritol phosphate (MEP) pathways [73]. MEP pathway starts with the condensation of pyruvate and D-glycerldehyde-3-phosphate derived respectively from glycolysis and the pentose phosphate pathway (PPP). The MVA pathway is initiated by the condensation of three molecules of acetyl-CoA (derived from pyruvate) with the 3-hydroxy-3-methylglutaryl-CoA [74]. Benzenoids synthesis starts by the shikimate pathway which precursors are phosphoenolpyruvate and D-erythrose 4-phosphate, also provided respectively from glycolysis and PPP pathways. Therefore, the same metabolic routes provide precursors for the MEP and MVA pathways, so that they have to compete with the shikimate pathway [75, 76]. This competition for the substrate could explain the VOCs profile observed in CMe-CPI.3 transgenic plants. While benzenoids synthesis is privileged, terpenoids emission is reduced. The rate of a VOC is not only controlled by the amount of the enzymes involved in its formation, but is rather conditioned by the availability of its substrate [77–79]. Precursor availability is also known to play a key role in the modulation of VOCs rhythmic emission [80–82] as plants emit volatiles with different diurnal and nocturnal patterns [83–85].

## Conclusions

In this study, we conferred resistance against *T. absoluta* to tomato plants by expressing two PIs from different mechanistic classes. The two PIs showed additive effect. Better efficiency was achieved when both genes were co-expressed. This direct noxious effect on *T. absoluta* was complemented by the attraction of its predator, *N. tenuis*. It is worthy to mention that the obtained transgenic plants had no detrimental effects on the mirid.

Barley cystatin Hv-CPI2 expression in tomato plants induced endogenous defensive response by activating *Pin2* gene, increasing glandular trichomes production and modifying VOCs emission. The harm occasioned to *T. absoluta* could though be caused not only by the direct effect of the foreign PIs, but also by the activation of tomato endogenous defensive response. The mechanism resulting in this activation remains to be elucidated. As far as we know, no previous study has reported the effect of the expression of a foreign cystatin on the host plant endogenous defense.

## Additional files

Additional file 1: Table. Primers used in this work (DOCX 11 kb)

Additional file 2: (.tif). Semi-quantitative PCR for *Icy* and *Itr1* genes in the homozygous plants. (a). Semi-quantitative PCR of *Icy2* gene; (b). Semi-quantitative PCR of *Itr1* gene; (c). Semi-quantitative PCR of the constitutive gene *SlActin*. (TIFF 103 kb)

Additional file 3: (.tif). Enzyme histochemistry of a *Tuta absoluta* L3 larvae cryocut. (a). Larval section incubated with BAAMC florescent substrate. (b). Negative control: larval section without BAAMC substrate. Proteases are localized along the digestive tract: Esophagus (Es), Foregut (Fg), Midgut (Mg), Hindgut (Hg), Malpighi tubules (Mt) and Exoskeleton (Ex). (TIFF 2353 kb)

## Abbreviations

BAAMC: Nα-Benzoyl-L-arginine-7-amido-4-methylcoumarin hydrochloride
BApNa: *N*_α_-Benzoyl-L-arginine 4-nitroanilide hydrochloride
CaMV: Cauliflower mosaic virus
Cv: cultivar
LB: left border
MEP: Methyl erythritol phosphate
MVA: Mevalonic acid
*nptII*: neomycin phosphotransferase II
P35S: Cauliflower mosaic virus 35S promoter
PFLNA: pGlu-Phe-Leu p-nitroanilide
PI: proteinase inhibitor
PNOS: Nopaline synthase promoter
PPP: Pentose phosphate pathway
PVA: Polyvinyl alcohol
RB: right border
Rv: reduction value
T35S: Cauliflower mosaic virus 35S terminator
TNOS: Nopaline synthase terminator
VOCs: Volatile organic compounds
