# Supplementary material for: Expression of two barley proteinase inhibitors in tomato promotes endogenous defensive response and enhances resistance to Tuta absoluta
Source: BMC Plant Biol. 2018 Jan 25;18:24. doi: 10.1186/s12870-018-1240-6 (PMC5785808; doi:10.1186/s12870-018-1240-6)
Supplement: Supplementary file 1 — Table. Primers used in this work (DOCX 11 kb) [file 12870_2018_1240_MOESM1_ESM.docx]

| **Primer name** | **Primer sequence** |
| --- | --- |
| CMeT S | ATGTTCGGGGATATGTGTGCT |
| CMeT AS | TTACAAGACCACTTCATATCC |
| T35SF-Spe | ACTGACTAGTTGTGATATCCCGCGGCCAT |
| T35SR-Sal | ACTGGTCGACGCAGGTCACTGGATTTTGGT |
| P35SF-Sph-Sal | ACTGGCATGCACGTCGAC CAAGCTGATCTCCTTTGCCCC |
| P35SR-SacII | ACTGCCGCGGCCGGAGTCCTCTCCAAATGA |
| HvItr1qF2 | GTATGTGTGCTCCAGGGGA |
| HvItr1qR2 | AGGTGGATAGTCTAGGGCCT |
| HvIcy2qF2 | GTCTCCGACCACAACAAGAAG |
| HvIcy2qR2 | ACATTTCTCGAACTCCAGCAG |
| SlActin8-F | CAAGTTATTACCATTGGTGCTGAGA |
| SlActin8-R | TGCAGCTTCCATACCAATCATG |
| Kan-dir | GAC AAG CCG TTT TAC GTT TG |
| Kan-rev | GAT ACT TTC TCG GCA GGA G |
