# Supplementary figures and images for: Expression of two barley proteinase inhibitors in tomato promotes endogenous defensive response and enhances resistance to Tuta absoluta
Source: BMC Plant Biol. 2018 Jan 25;18:24. doi: 10.1186/s12870-018-1240-6 (PMC5785808; doi:10.1186/s12870-018-1240-6)

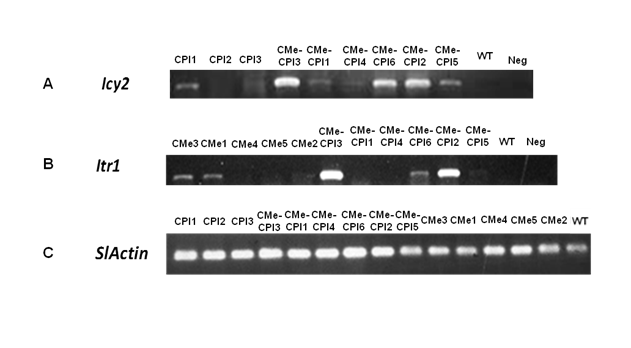

Supplement: Supplementary file 2 — (.tif). Semi-quantitative PCR for Icy and Itr1 genes in the homozygous plants. (a). Semi-quantitative PCR of Icy2 gene; (b). Semi-quantitative PCR of Itr1 gene; (c). Semi-quantitative PCR of the constitutive gene SlActin. (TIFF 103 kb) [file 12870_2018_1240_MOESM2_ESM.tif]

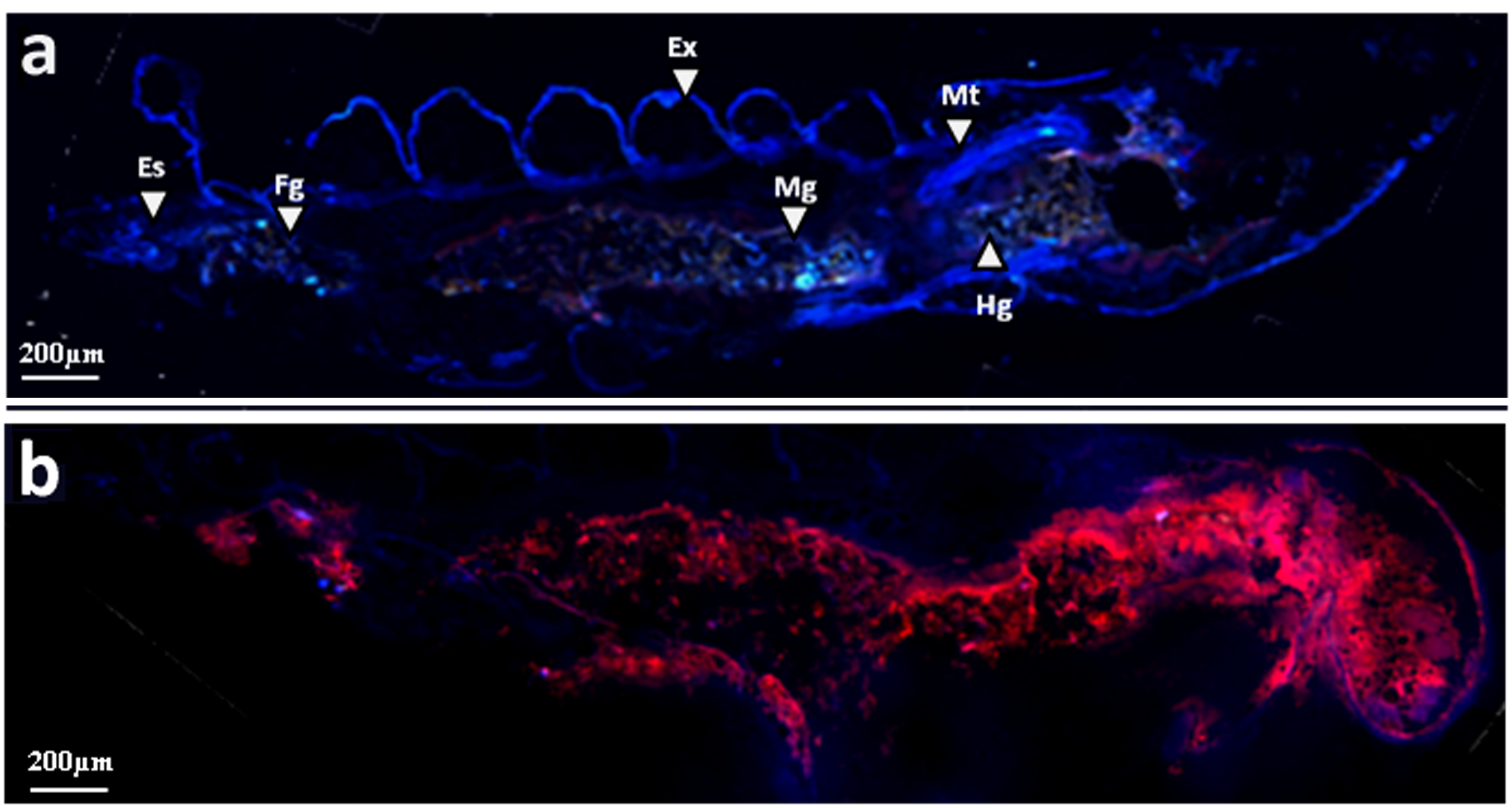

Supplement: Supplementary file 3 — (.tif). Enzyme histochemistry of a Tuta absoluta L3 larvae cryocut. (a). Larval section incubated with BAAMC florescent substrate. (b). Negative control: larval section without BAAMC substrate. Proteases are localized along the digestive tract: Esophagus (Es), Foregut (Fg), Midgut (Mg), Hindgut (Hg), Malpighi tubules (Mt) and Exoskeleton (Ex). (TIFF 2353 kb) [file 12870_2018_1240_MOESM3_ESM.tif]
